# Linear and nonlinear investigations for the adsorption of paracetamol and metformin from water on acid-treated clay

**DOI:** 10.1038/s41598-021-93040-y

**Published:** 2021-06-30

**Authors:** Mohamed R. Elamin, Babiker Y. Abdulkhair, Faisal K. Algethami, L. Khezami

**Affiliations:** 1 College of Science, Chemistry Department, Imam Mohammad Ibn Saud Islamic University (IMSIU), P.O. Box 90905, Riyadh, 11623 Kingdom of Saudi Arabia; 2 Industrial Research and Consultancy Center (IRCC), Khartoum North, Sudan; 3 College of Science, Chemistry Department, Sudan University of Science and Technology (SUST), Khartoum, Sudan

**Keywords:** Environmental sciences, Chemistry

## Abstract

Natural clays are considered a safe, low-cost, and sound sorbent for some pharmaceutical and body care products from water. Metformin (MF) and paracetamol (PA) are of the most consumable drugs worldwide. A portion of natural clay was treated with distilled water, and another part was treated with hydrochloric acid. The water-treated clay (WTC) and the acid-treated clay (ATC) were characterized by scanning electron microscopy-energy dispersive spectroscopy, X-ray diffraction, Fourier transforms infrared spectroscopy, and nitrogen adsorption isotherm. Batch experiments were employed to investigate the influence of contact time and solution parameters on the adsorption of PA and MF on WTC and ATC. 30 min attained the equilibrium for all sorbent-sorbate systems. Both sorbents fitted the pseudo-second-order kinetic model with a preference to the nonlinear fitting, and the mechanism of adsorption partially fitted the liquid-film diffusion model. The PA and MF adsorption on WTC and ATC fitted the Freundlich model in preference to nonlinear fitting. The adsorption of pollutants on both sorbents was spontaneous, exothermic, and physisorption in nature. Even at low concentrations, both WTC and ATC showed efficiency above 80% in removing PA and MF from tab water, groundwater, and Red seawater. These findings nominated natural clay as an alternative to the costly nanomaterials as sorbents for removing pharmaceutical contaminants from water.

## Introduction

The presence of organic pollutants in water systems is a devastating problem facing the global environment these days. The growing demand and intensive use of Pharmaceuticals and personal care products (PPCPs) lead to the accumulation of their residues in water and wastewater systems^1,2^. These PPCPs may enter the water systems from humans and animals urine and feces, expired drugs discarded, washing of machinery, and discarding improperly manufactured pharmaceuticals^3–5^. The availability of PPCPs in water systems may cause epidemic bacteria and other microbes to gain immunity against these drugs on long exposure periods, leading to the evolution of drug resisting microbes, and finally, treatment failure^6,7^. Different illumination practices were encountered to remove the PPCPs residues from wastewater, including oxidation, coagulation-precipitation, reverse osmosis, electro-dialysis, and ion exchange^8–11^. The high cost of these processes and the need for continuous monitoring are considered among the main disadvantages of these technologies^12^. Adsorption is a simple procedure that can be carried out with low-cost sorbents like carbonaceous materials or natural clay minerals^13–20^. Although carbon materials have a large surface area, clay materials can be free because they are found naturally. Among all known adsorbents used in water treatment, clay is the cheapest and safest to the degree that it can be used as excipients or active substances in many pharmaceutical products^21–29^. Metformin (MF) and paracetamol (PA) are among the most consumable drugs worldwide^30–32^. PA was one of the eighteen PPCPs found in a Shallow lake—China, while both MF and PA were of the thirteen PPCPs found in the red-sea water^33,34^. In addition, PA consumption is expected to increase enormously as it had been recommended to treat the symptoms of the COVID-19 pandemic^35–39^. Natural clay is a safe and low-cost material, making it a suitable alternative to nano-sorbents if it has an adequate adsorption capacity. a natural clay NC was collected from the Eldoushain area, Nile river state, Sudan (16°45′43.1″N 33°35′25.0″E)^40–43^. To improve the adsorption capacity, water treated clay (WTC) and an acid-treated clay ATC were employed for the removal of MF and PA from real water samples. Since some recent papers have recommended the employment of nonlinear fitting, herein, in this study both the linear and the nonlinear fittings were employed to determine the best on a statistical basis^44–49^.

## Results and discussion

### Characterization of WTC and ATC

**Figure 1:**
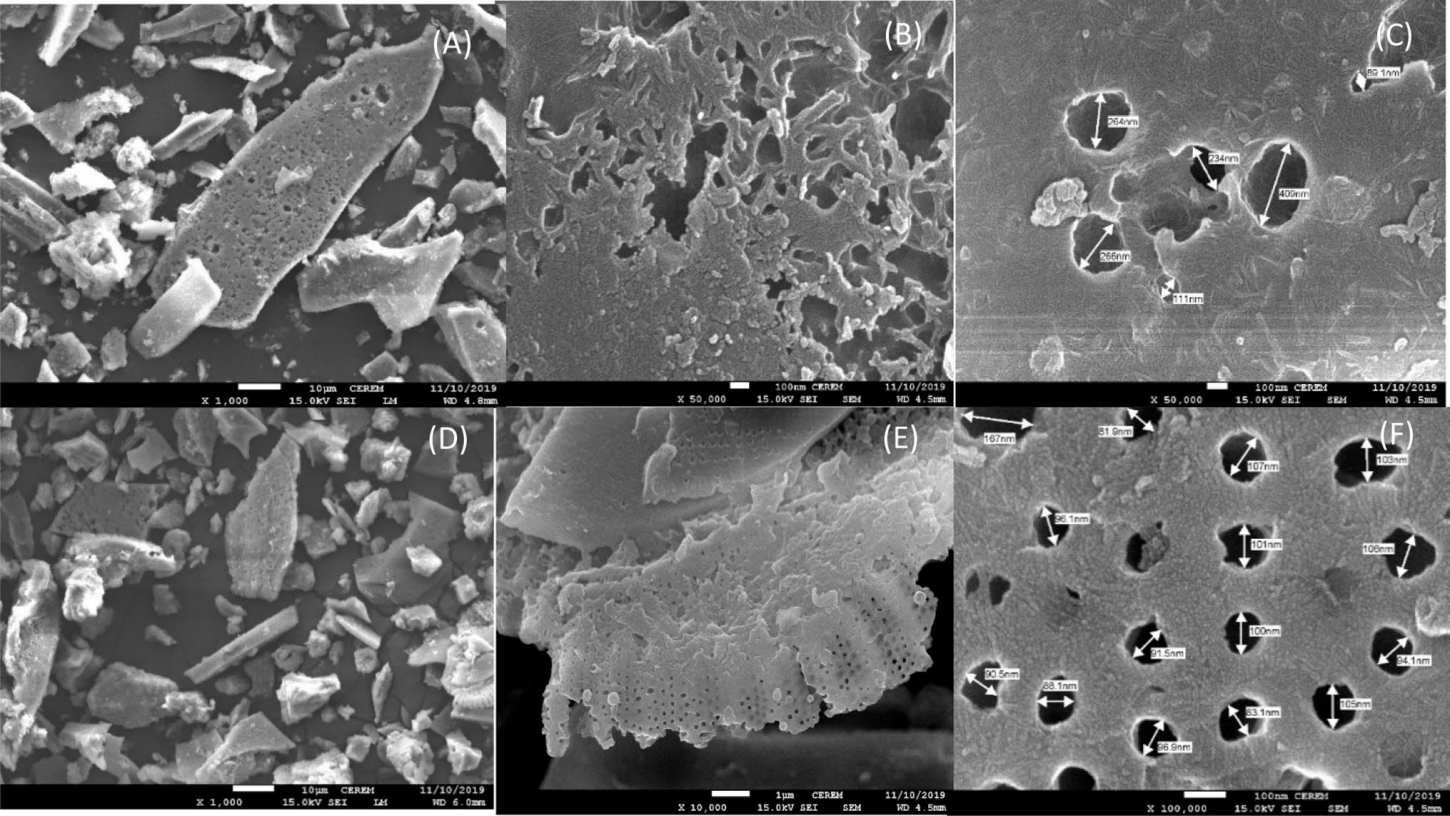
SEM images of the clay WTC (**a**–**c**), ATC (**d**–**f**).

**Figure 2:**
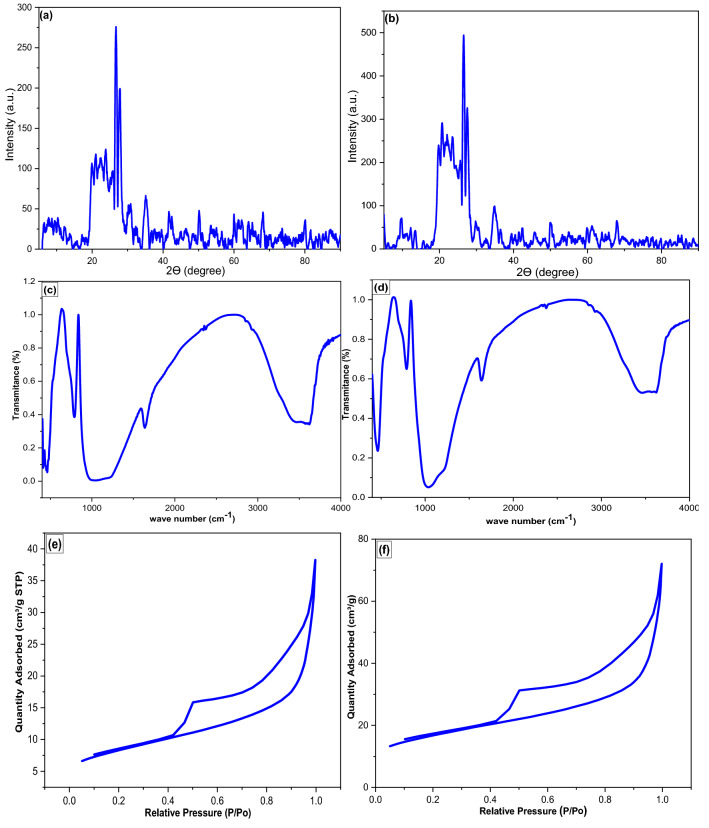
(**a**,**b**) XRD pattern of WTC and ATC, respectively; (**c**,**d**) are the FTIR results of WTC and ATC, respectively; (**e**,**f**) are the N2 adsorption–desorption isotherms results of WTC and ATC, respectively.

The surface topography of WTC and ATC was scanned with SEM. The low magnification of the WTC shows flakes with some cavities on their surface as monitored in Fig. 1a. The medium magnification revealed the irregular shape and size of the cavities which may be due to debris and mineral crystals (Fig. 1b). High magnification revealed a surface coated with fine nano granules with pores of 89–409 nm diameters (Fig. 1c). For the ATC sample, although the low magnification reflected no difference for the acid treatment (Fig. 1d), the medium magnification showed an efficient removal of debris-cover revealing a diatomaceous structure (Fig. 1e). Figure 1f illustrated relatively regular pores with a 100 nm average diameter. The porous nature of this material imparts a high surface area and porosity, making it suitable for adsorption and filtration. These results are consistent with that previously reported^50–52^. The elemental analysis of WTC and ATC was carried out using EDS. For WTC the content of silicon, aluminum, sodium, and potassium was 64.4%, 8.96%, 3.25%, and 1.92%, respectively; while ATC appeared to contain 72.5%, 9.12%, 2.56%, and 1.73%, respectively. These results confirmed that a considerable portion of the samples is of diatomaceous earth composition (SiO_2_). The sodium, potassium, and iron were found in both samples, although the contents are a little bit lower in the ATC due to the acid treatment. Figure 2a, b demonstrates the XRD patterns of WTC and ATC, respectively. The main diffraction peaks, in both sorbents, are the diffraction peaks of SiO_2_, confirming the existence of diatomaceous earth mainly located in the 2*θ* range of 20°–40°. The diffraction peaks with 2*θ* values of 21.0, 35.4 for WTC and 20.8 and 34.8 for ATC, can be assigned to (101) and (112) crystal faces of SiO_2_ (JCPDS Card No. 82-0512). In the pattern of WTC, the peaks at 2*θ* of 8.0, 10.4, 12.3, 26.7, 27.82, 35.4, 37.3, 50.1, and 54.3 are assigned to the various types of clay minerals. The ATC pattern showed peaks at 2*θ* of 5.17, 9.83, 19.7, 20.8, 26.72, 27.8, 25.5, 34.8, 36.35. 39.45, 43.5. 50.1, 58.37, and 68. These results were similar to the results previously reported^27^. The FTIR spectra for WTC and ATC are shown in Fig. 2c, d, respectively. The WTC showed six peaks at 461.7 cm^−1^ (s), assigned to Si–O–Si bending vibrations, 793.7 cm^−1^, for Si–O–Al vibrations, 1022 cm^−1^ (s) for Si–O stretching, indicating the existence of montmorillonite clay. The peaks at 1645 cm^−1^ and 3412 cm^−1^ are assigned to bending and stretching vibrations of the combined water, respectively. The peak at 3660 cm^−1^ can be attributed to the stretching vibrations of the Si–Si–OH and/or Si–Al–OH. The vibration bands for ATC are similar to those of WTC with slight differences in the absorption of some bands, appearing at 461.7, 793.7, 1022 (s), 1666, 3453, and 3640 cm^−1^. The spectra of FTIR for WTC and ATC were found to be similar to those previously reported for clays^50,52^.

BET surface area of the sorbents was determined; for instance, Fig. 2e, f showed the evolution of N_2_ adsorption–desorption isotherms for WTC and ATC, respectively. Both WTC and ATC exhibited a sorption isotherm of type IV(a), with a hysteresis of H4 type^53^. Such hysteresis type is an indicator of narrow slit-like pores, particles with internal voids of irregular shape and broad size distribution, hollow spheres with walls composed of ordered mesoporous particles. At elevated relative pressure *P*/*P*^o^, the hysteresis of H4 type was due to the filling up of mesopores by capillary condensation, indicating a shape of pores that was flatter instead of cylindrical. The Surface area of WTC and ATC was 9.53 and 11.49 m^2^ g^−1^, respectively. The pore diameter for WTC and ATC was determined by the BJH method; the average diameter of pores decreased from 8.64 to 8.39 nm. Inversely, the pore volume was increased from 2.03 × 10^–2^ to 2.22 × 10^–2^ cm^3^ g^−1^. These results confirmed the improvement implemented by acid treatment since the surface area increased by about 2.0 m^2^ g^−1^.

### Adsorption studies

Adsorption of MF and PA in an aqueous solution by WTC and ATC was studied. The adsorption was possibly due to Van der Waals interactions and/or electrostatic attraction between the pharmaceutical pollutant and the sorbents^54–56^. The impact of WTC and ATC mass on the trend of adsorption for MF and PA was investigated. As the sorption sites increase by increasing the mass of sorbent, the adsorption percentage increased; meanwhile, the adsorption capacity decreased inversely. The efficiency of WTC and ATC in the removal of PA and MF are comparable to those found in the literature with the advantage of low cost for the sorbent in this study^57–63^.

The contact time study revealed that 20 min sufficed the adsorption of MF and PA on ATC to reach the equilibrium; on the other hand, it takes 30 min for the adsorption of PA and MF on WTC (S. 1). The impact of ionic strength on the adsorption of PA and MF on WTC and ATC was investigated. The decrease in adsorption efficiency as the ionic strength increased may be attributed to the influence of salinity on the adsorption process (S. 2a). Also, the significant reduction in the adsorption efficiency of the MF may be attributed to the five partially negative nitrogen atoms that may repulse with any negative sites on the sorbent surface and/or negative sorbates^64,65^. The pH effect on the adsorption of PA and MF on WTC and ATC was investigated. The best adsorption percentage for MF on both WTC and ATC was achieved at a pH value of 4.0; on the other hand, the best adsorption percentage for PA on both WTC and ATC was achieved at a pH value of 6.0 (S. 2b). Moreover, the influence of initial fed concentrations of PA and MF on WTC and ATC adsorption efficiency was studied. The adsorption percentage for both sorbents was inversely proportional to the initial fed concentration for both drugs. This trend illustrates that both sorbents can be very effective for treating MF and PA at low concentrations, as expected to be in the polluted water resources.

### Adsorption kinetic order

The some studies in the literature highlighted the misleading results of the linear pseudo-second-order kinetic equations^66^. Considering that, non-linear pseudo-first-order (NLPFO) (Eq. 1), non-linear pseudo-second-order (NLPSO) (Eq. 3), linear pseudo-first-order (LPFO) (Eq. 2), and linear pseudo-second-order (LPSO) (Eq. 4) were all employed for the kinetic study investigations^67–69^.

1 $$ q_{t} ~ = ~q_{e} \left( {1~ - ~exp^{{ - K_{{1.}} t}} ~} \right) $$

2 $$ ln\left( {q_{e}  - ~q_{t} } \right) = \ln q_{e}  - ~k_{1}  \cdot t $$

3 $$ ~q_{t}  = ~\frac{{k_{2}  \cdot q_{e}^{2}  \cdot t}}{{1 + k_{2}  \cdot q_{e}  \cdot t}} $$

4 $$ \frac{t}{{q_{t} }} = ~\frac{1}{{k_{2}  \cdot q_{e}^{2} }} + ~\frac{t}{{q_{e} }}~ $$

where k_1_(min^–1^) is the PFO rate constant, k_2_ (g mg^–1^ min^–1^) is the PSO rate constant, q_t_ and q_e_ (mg g^-1^) are the adsorbed mass of drug per unit mass of sorbent at time t and equilibrium, respectively.

The adsorption rate constant were calculated from the slope of the corresponding regression^70^.

**Figure 3:**
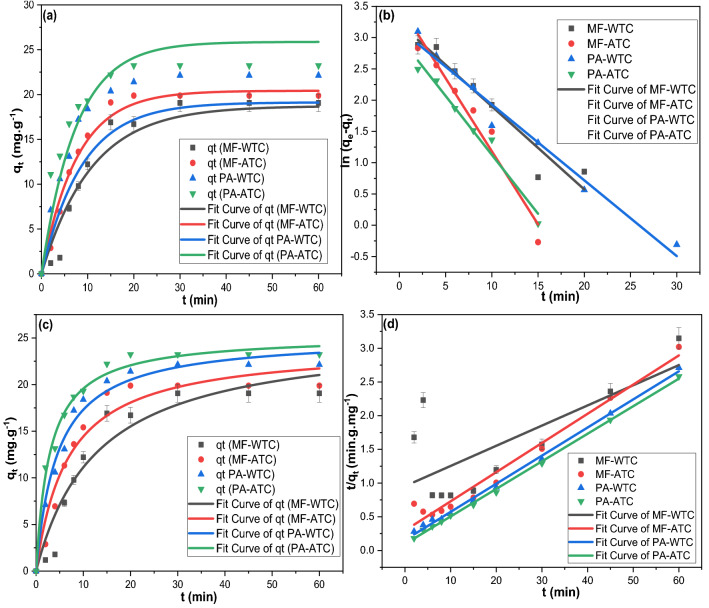
(**a**) NLPFO, (**b**) LPFO, (**c**) NLPSO, and (**d**) LPSO fittings for the adsorption of MF and PA on WTC and ATC.

The adsorption kinetic orders were determined by the best linear fit judged usually by the best correlation coefficient (R^2^) value^66^. Also, the residual sum of squares (RSS), the value of Chi-squared (*χ*^2^), were considered as they reflects the goodness of regressions (Fig. 3; Table 1). The adsorption of MF and PA on WTC and ATC fitted the PSO kinetic models since it revealed the lowest *χ*^2^, the highest R^2^, a semi-typical q_e,_ and the lowest RSS value. Worthmentioning that the q_e_ predicted from the NLPSO was almost typical to the experimental values for all sorbent–sorbate systems in this study.

**Table 1:** The kinetics and isotherms parameters for the adsorption of MF and PA on WTC and ATC.

Parameter ↓	Sorbent-sorbate	LPFO	NLPFO	LPSO	NLPSO	Sorbent-sorbate	LPFO	NLPFO	LPSO	NLPSO
*Adsorption kinetic models*										
q_e_ exp. (mg.g^-1^)	WTC-MF	19.061				ATC-MF	22.133			
q_e_ cal. (mg/g)		25.268	18.719	33.411	25.582		33.788	20.426	23.095	24.167
Rate constant		0.133	0.093	0.001	0.003		0.234	0.131	0.006	0.006
R^2^		0.927	0.939	0.515	0.927		0.957	0.984	0.971	0.951
*X*^*2*^		0.019	3.524	0.355	4.688		0.059	0.962	0.024	2.928
RSS		0.336	31.715	2.838	42.189		0.264	8.656	0.192	26.353
q_e_ exp. (mg/g)	WTC-PA	19.879				ATC-PA	23.233			
q_e_ cal. (mg/g)		23.136	19.127	23.969	25.186		20.289	25.880	24.450	30.523
Rate constant		0.121	0.114	0.011	0.009		0.188	0.134	0.017	0.005
R^2^		0.977	0.936	0.997	0.982		0.973	0.967	0.998	0.988
*X*^*2*^		0.046	3.890	0.002	1.110		0.110	3.231	0.001	0.722
RSS		0.215	35.009	0.020	9.989		0.105	29.082	0.009	6.500

Sorbate -sorbent ↓	IPDM					LFDM				
	K_IP_ (mg.g^-1^ min^1/2^)	C (mg. g^-1^)	*R*^*2*^	X^2^	RSS	K_LF_ (min–1)	R^2^	X^2^	RSS	
*Adsorption rate-control mechanism models*										
MF-WTC	2.968	0.398	0.775	12.448	99.585	0.133	0.927	0.067	0.336	
MF-ATC	2.437	5.101	0.682	13.498	107.987	0.234	0.957	0.066	0.264	
PA-WTC	2.192	8.660	0.708	9.629	77.034	0.170	0.994	0.009	0.045	
PA-ATC	1.819	12.115	0.707	6.664	53.312	0.188	0.973	0.026	0.105	

	MF-WTC	MF-WTC	PA-WTC	PA-ATC	MF-WTC	MF-WTC	PA-WTC	PA-ATC		
*Adsorption Isotherms parameters*										
	LLM				NLLM					
q_m_ (mg g^−1^)	31.827	37.453	43.917	25.240	36.343	29.357	46.110	32.673		
K_L_	0.175	0.191	0.085	0.990	0.004	0.011	0.003	0.015		
X^2^	0.000	0.001	0.009	0.000	0.263	0.454	0.904	2.277		
RSS	0.001	0.002	0.028	0.001	0.789	1.361	2.711	6.832		
R^2^	0.969	0.858	0.314	0.930	0.995	0.993	0.987	0.972		
C_o_ ↓	S_L_ values from LLM				S_L_ values from NLLM					
1	0.851	0.840	0.922	0.503	0.996	0.989	0.997	0.985		
5	0.533	0.512	0.702	0.168	0.981	0.949	0.985	0.931		
10	0.363	0.344	0.541	0.092	0.963	0.903	0.971	0.870		
15	0.275	0.259	0.440	0.063	0.946	0.861	0.958	0.817		
20	0.222	0.208	0.370	0.048	0.929	0.823	0.944	0.770		
	LFM				NLFM					
K_F_	4.269	5.700	5.088	10.411	1.433	1.648	1.352	1.779		
n	0.799	0.816	0.860	0.910	4.793	6.921	5.752	10.528		
X^2^	0.016	0.044	0.017	0.024	0.105	1.364	1.143	0.491		
RSS	0.048	0.131	0.051	0.073	0.316	4.092	3.428	1.473		
R^2^	0.991	0.975	0.991	0.986	0.998	0.979	0.983	0.994		

### Adsorption rate-controlling mechanism

The adsorption rate-control-mechanism was investigated for the adsorption of PA and MF on WTC and ATC by the employment of the intra-particle diffusion model (IPDM) (Eq. 5) and the liquid film-diffusion model (LFDM) (Eq. 6)^67,71^.

5 $$ q_{t}  = K_{{ip}} *t^{{\frac{1}{2}}}  + C_{i} $$

where k_ip_ (mg g^−1^ min^-0.5^) is the rate constant of the IPDM. C_i_: concentration in mg g^-1^ (parameter relates to the boundary layer thickness).

6 $$ ln\left( {1 - F} \right) = ~ - K_{{LF}} *t $$

where

$$ {\text{F}} = {\text{q}}_{{\text{e}}} {\text{/q}}_{{\text{t}}} $$

k_LF_ (min^–1^) is the equilibrium fractional attainment.

**Figure 4:**
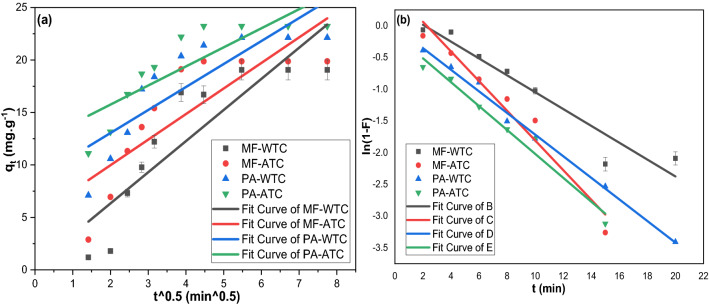
(**a**) IPDM, and (**b**) LFDM fittings for the adsorption of MF and PA on WTC and ATC.

Although the K_IP_ values were much more than the K_LF_ values indicating the preference of IPDM, it worth mentioning that the high C_i_ values indicated a high boundary-layer resistance to the IPDM^72^. The regression results of LFDM and IPDM fittings (Fig. 4) revealed that LFDM mainly controlled the adsorption of PA and MF on WTC and ATC since it had the least RSS, the highest R^2^, and the least *χ*^2^ as demonstrated in Table 1. Besides, the multi-linearity curve produced by IPDM evident that the adsorptions of PA and MF on WTC and ATC were complicated ones that may be controlled by two or more mechanisms^73,74^. This complexity may be attributed to the diverse nature of the clay sorbent; the presence of diverse types of clay minerals may impart to the difference in diffusion models.

### Adsorption isotherms

The Langmuir and Freundlich isotherms were employed to investigate the adsorption of PA and MF on WTC and ATC. The linear and nonlinear forms of the Langmuir model (LLM and NLLM) were utilized to investigate the monolayer adsorption with no sorbate penetration in the surface-plane explained by Eqs. (7) and (8), respectively. The adsorption nature was further investigated by the Langmuir isotherm separation factor (S_L_) (Eq. 9).

7 $$ \frac{{C_{e} }}{{q_{e} }} = ~\frac{1}{{K_{L}  \cdot q_{m} }} + \frac{{C_{e} }}{{q_{m} }} $$

8 $$ q_{e}  = ~\left( {\frac{{K_{l} q_{m} C_{e} }}{{1 + q_{m} C_{e} ~}}} \right) $$

9 $$ S_{L} {\text{~}} = {\text{~}}\frac{1}{{1 + K_{L} {\text{~}} \cdot C_{o} }} $$

where C_e_ is the drug concentration at equilibrium, q_m_ and K_L_ (L mg^−1^) represent the maximum adsorption capacity, and the Langmuir constant corresponds to the monolayer adsorption, respectively. As a multilayered adsorption model, the linear and the nonlinear forms of the Freundlich model (LFM and NLFM) were employed in this study (Eqs. 10, 11, respectively).

10 $$ ~\ln q_{e}  = ~lnK_{F}  + ~\frac{1}{n}\ln c_{e} ~ $$

11 $$ q_{e}  = ~K_{F} ~ \cdot C_{e}^{{{\raise0.7ex\hbox{$1$} \!\mathord{\left/ {\vphantom {1 n}}\right.\kern-\nulldelimiterspace} \!\lower0.7ex\hbox{$n$}}}} $$

**Figure 5:**
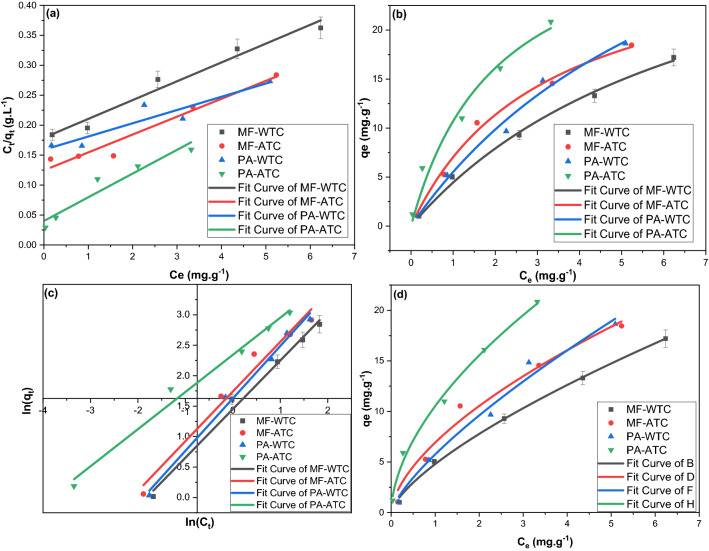
(**a**) LLM, (**b**) NLLM, (**c**) LFM and (**d**) NLFM for the adsorption of PA and MF on WTC and ATC.

*K*_*F*_ (L g^−1^) is the Freundlich constant, while ***n*** (arbitrary) is the Freundlich heterogeneity factor related to the adsorption capacity. Worth mentioning that an n value ranged between 0 and 10 indicates the favourability adsorption process^75^. Figure 5 illustrated the fitted curves of isotherms while the obtained isotherms parameters were monitored in Table 1. The adsorption of PA and MF on WTC and ATC does not fit the LLM or NLLM. Meanwhile, the S_L_ results calculated from the K_L_ generated by both models reflected the favorable nature of the adsorption process (S_L_ values less than one)^76^. So, based on the obtained statistical parameters, the adsorption of PA and MF on WTC and ATC fitted the NLFM with the highest R^2^ values and the least RSS and χ^2^ values. Besides, the n values generated from NLFM revealed a strong adsorption bond since the n values ranged between 4.793 and 10.528^77–80^.

## Thermodynamic studies

The temperature impact on the adsorptions of PA and MF on WTC and ATC was investigated, and for a better understanding of the adsorption behaviors of PA and MF on WTC and ATC, the thermodynamics of this process was investigated. The thermodynamic parameters, including the enthalpy (ΔH^o^), entropy (ΔS^o^), and Gibbs free energy (ΔG^o^), were evaluated (Eqs. 12, 13).

12 $$ \ln K_{c}  = ~\frac{{\Delta H^{{~o}} }}{{RT}}~ + ~\frac{{\Delta S~^{o} }}{R} $$

13 $$ \Delta ~G^{{~o}}  = ~\Delta ~H^{{~o}}  - T~\Delta ~S~^{o} ~ $$

**Figure 6:**
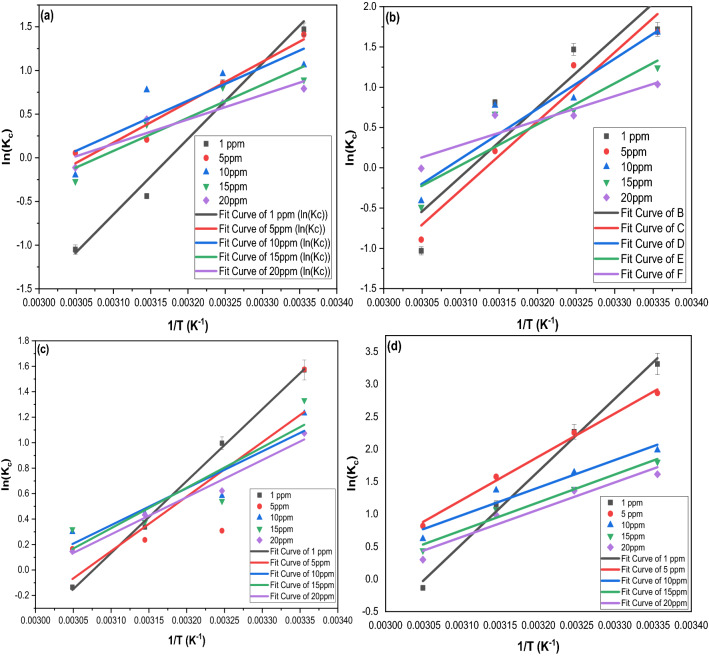
The plot of ln *K*_c_ against 1/*T* for the adsorption of different initial feed concentrations of (**a**) MF on WTC, (**b**) MF on ATC, (**c**) MF on WTC, and (**d**) MF on ATC.

The ΔG^o^, ΔH^o^, and ΔS^o^ (kJ mol^−1^) were calculated from the plot of ln(K_c_) versus (1/T) as shown in Fig. 6. The ideal gas constant (R = 0.0081345 kJ mol^−1^) was applied in all calculations, and the thermodynamic parameters were monitored in Table 2. The negative ΔH^o^ values indicated the exothermic nature of the adsorption of PA and MF on both sorbents meaning that the adsorption efficiency may be enhanced by lowering the solution’s temperature. Besides, a ΔH^o^ of less than 40 kJ mol indicates a physisorption process; conversely, it is considered chemisorption when having a ΔH^o^ of 80–450 kJ/mol^81–83^. The adsorption spontaneity was inferred from the negative ΔG^o^ values for both pollutants on WTA and ATC^84^; this finding was supported by the negative ΔS^o^ values^85^. Also, the adsorption appeared to go less spontaneous as the temperature increased. Nevertheless, the obtained ΔG^o^ values (less than 20 kJ mol^−1^) indicated that the adsorption process is a physisorption^67,68,83,86–88^.

**Table 2:** Thermodynamic parameters for the adsorption of MF and PA on WTC and ATC at a temperature range of 298–328 ^o^K.

Fed conc (mg L^−^1)	ΔH^o^ (kJ mol^−1^)	ΔS^o^ (kJ mol^−1^)	ΔG^o^ (kJ mol^−1^) 298 K	ΔG^o^ (kJ mol^−1^) 308 K	ΔG^o^ (kJ mol^−1^) 318 K	ΔG^o^ (kJ mol^−1^) 328 K
*Thermodynamic parameters*						
**MF-WTC**						
1	− 71.902	− 0.228	− 3.873	− 1.591	0.692	2.975
5	− 38.462	− 0.118	− 3.361	− 2.183	− 1.006	0.172
10	− 31.795	− 0.096	− 3.096	− 2.133	− 1.170	− 0.207
15	− 31.477	− 0.097	− 2.594	− 1.625	− 0.656	0.313
20	− 23.351	− 0.071	− 2.171	− 1.461	− 0.750	− 0.039
**MF-ATC**						
1	− 71.532	− 0.223	− 5.179	− 2.953	− 0.726	1.501
5	− 71.110	− 0.223	− 4.731	− 2.504	− 0.276	1.951
10	− 51.562	− 0.159	− 4.210	− 2.621	− 1.032	0.557
15	− 42.236	− 0.131	− 3.299	− 1.992	− 0.686	0.621
20	− 25.248	− 0.076	− 2.624	− 1.865	− 1.106	− 0.346
**PA-ATC**						
1	− 46.981	− 0.145	− 3.910	− 2.464	− 1.019	0.426
5	− 35.564	− 0.109	− 3.078	− 1.988	− 0.897	0.193
10	− 24.079	− 0.072	− 2.708	− 1.991	− 1.274	− 0.557
15	− 26.420	− 0.079	− 2.826	− 2.034	− 1.243	− 0.451
20	− 24.243	− 0.073	− 2.542	− 1.814	− 1.086	− 0.357
**PA-WTC**						
1	− 92.922	− 0.284	− 8.424	− 5.589	− 2.753	0.082
5	− 55.273	− 0.161	− 7.235	− 5.623	− 4.011	− 2.399
10	− 35.263	− 0.101	− 5.127	− 4.116	− 3.104	− 2.093
15	− 35.675	− 0.104	− 4.583	− 3.540	− 2.497	− 1.453
20	− 34.836	− 0.103	− 4.267	− 3.241	− 2.216	− 1.190

### Application to natural water samples

Tap water (TW) was collected from the chemistry Lab at Riyadh, Kingdom of Saudi Arabia (KSA). The Red Sea water sample (RSW) was picked from the coast of Jeddah City—KSA, while the groundwater (GW) sample was collected from the Sudair area (150 km North Riyadh—KSA). All samples were collected during the summer season, the total dissolved salts for TW, GW, and RSW was 0.2, 1.1 and 32.5 g L^−1^, respectively; and the total hardness was 146.2, 751.8 and 7293 mg L^−1^, respectively. The applicability of WTC and ATC for the removal of MF and PA from TW, GW, and RSW was explored. A concentration of 1, 5, and 10 mg L^−1^ were prepared for each drug in 250 mL of each water sample. The optimized conditions were applied, and the removal of PA and MF by WTC and ATC (S. 3). WTC and ATC were found to be efficient for the removal of PA and MF from the three samples. Besides, a promising result has emerged during this application that the total dissolved solids (TDS) of RSW, which was measured by a conductivity-meter (Jennway 6605), decreased significantly after being in contact with the sorbents revealing a desalination capacity of 1200 and 1500 mg g^−1^ for WTC and ATC, respectively.

## Conclusion

This work demonstrates the optimization of adsorption of PA and MF by WTC and ATC from aqueous solution. The optimized conditions were applied to real environmental water samples. The adsorption isotherms data fitted the Freundlich model, and the adsorption processes obeyed the pseudo-second-order with a better regression parameter with the NLPSO. The maximum adsorption capacity ranged between 20.4 and 30.5 mg g^−1^. The application of both sorbents for fast removal of PA and MF from contaminated water is recommended. Considering the almost-zero-cost sorbent, the relatively short intake time and the desalination effect.

## Experimental

### Materials

NC was collected from the Eldoushain area, Nile River state, Sudan, hydrochloric acid 37% was purchased from Sharlau-Spain. The active pharmaceutical ingredients MF and PA were purchased from Ranbaxy Laboratories Limited, India. The washing of sorbent and the preparation of all solutions were performed using distilled water.

### Preparation of WTC and ATC

The NC was crushed in a porcelain mortar and milled by ball milling at 500 rpm for 30 min, the ball: clay mass was 1:1. 10 g of NC were stirred with 200 mL distilled water for 1 h, filtered through Buchner system, washed by 1 L distilled water, dried at 120 °C for 2 h, cooled in a desiccator, and kept in a polypropylene container. 10 g of NC were stirred with 200 mL of diluted hydrochloric acid (1:1), filtered through Buchner system, washed by distilled water (to pH = 7), dried at 120 °C for 2 h, cooled in a desiccator, and kept in a polypropylene container.

### Characterization of WTC and ATC

WTC and ATC were characterized using scanning electron-energy dispersive X-ray technique (SEM–EDS), model JSM-IT300. The WTC and ATC crystalline structures were examined by X-Ray diffractometer, Bruker, D8 Advance; Billerica, MA, USA. The functional groups for both sorbents were monitored using Bruker TENSOR-FTIR spectrophotometer (Germany). Characteristics of pores (i.e., pore diameter and volume) and specific Brunauer–Emmett–Teller (BET) surface area were evaluated based on N_2_ adsorption–desorption isotherms at 77 K using an ASAP 2020 Micromeritics analyzer. The surface area, pore size and pore diameter were calculated from the Brunauer–Emmett–Teller (BET) equation^89^.

### Adsorption experiments

Both PA and MF were analyzed by UV–Vis spectrophotometer (D3500 LABOMED) at 234 nm and 245 nm, respectively. The adsorption parameters were optimized, including the impacts of the initial fed concentration (1–20 mg L^−1^), sorbate–sorbent contact time, solution pH (1.0–10.0), and solution temperature (298–318 K). Also, the impact of ionic strength on the adsorption process was conducted by performing preparing pollutants solutions in sodium chloride solution(0.01–0.1 M). For the contact time study, 20.0 mg L^–1^ solution of each drug was prepared in distilled water with the aid of sonication. 80 mg of each sorbent was stirred with 200 mL of each solution, and 5 mL of each mixture was taken during the time; each solution was then filtered by a nylon syringe filter (0.22 µm) and subjected to UV–Vis analysis. The percentage of adsorption and adsorption capacity were calculated by Eqs. (14) and (15).

14 $$ ~{\text{Adsorption}}\,{\text{~percentage}} = ~\frac{{\left( {C_{o}  - ~C_{t} } \right)}}{{C_{o} }}~ \times 100\% $$

15 $$ q_{t}  = ~\frac{{\left( {C_{o}  - ~C_{t} } \right)~V}}{M}~, $$

where C_o_ is the initial fed concentration, C_t_ is the unabsorbed concentration, q_t_ is the adsorbed amount (mg g^−1^), V is the volume of the solution (L), and M is the mass of sorbent (g). Each experiment was carried three times, and the average was calculated.

## Supplementary Information

Supplementary Figures.
