# Supplementary material for: Linear and nonlinear investigations for the adsorption of paracetamol and metformin from water on acid-treated clay
Source: Sci Rep. 2021 Jun 30;11:13606. doi: 10.1038/s41598-021-93040-y (PMC8245496; doi:10.1038/s41598-021-93040-y)
Supplement: Supplementary file 1 — Supplementary Figures. [file 41598_2021_93040_MOESM1_ESM.docx]

**Supplementary**

**Linear and nonlinear investigations for the adsorption of paracetamol and metformin from water on acid-treated clay**

Mohamed R. Elamin^1,2^, Babiker Y. Abdulkhair^1,3*^, [Faisal K. Algethami](https://www.sciencedirect.com/science/article/abs/pii/S1010603019313279#!)^1^, L. Khezami^1^

^1^ Imam Mohammad Ibn Saud Islamic University (IMSIU), College of Science, Chemistry Department, Riyadh, KSA.

^2^ Industrial research and consultancy center (IRCC), Khartoum North, Sudan.

^3^ Sudan University of Science and Technology (SUST), College of Science, Chemistry Department, Khartoum, Sudan.

**Corresponding author**

Dr. Babiker Y. Abdulkhair

Chemistry Department - College of Science

Imam Mohammad Ibn Saud Islamic University

P.O. Box 90905, Riyadh, 11623, KSA

e-mail: [babiker35.by@gmail.com](mailto:babiker35.by@gmail.com)

byabdulkhair@imamu.edu.sa

**S. 1** Time influence on the adsorption of MF and PA by WTC and ATC.

**S. 2** The influence of (a) ionic strength and (b) solution pH on the adsorption of MF and PA by WTC and ATC from aqueous solutions at 25 ^o^C.

**S. 3** Removal of different concentrations of PA and MF by WTC and ATC from (a) TW, (b) GW, and (c) RSW at the optimized solution parameters.
